# Feasibility of using a World Health Organization-standard methodology for Sample Vital Registration with Verbal Autopsy (SAVVY) to report leading causes of death in Zambia: results of a pilot in four provinces, 2010

**DOI:** 10.1186/1478-7954-9-40

**Published:** 2011-08-05

**Authors:** Sheila S Mudenda, Stanley Kamocha, Robert Mswia, Martha Conkling, Palver Sikanyiti, Dara Potter, William C Mayaka, Melissa A Marx

**Affiliations:** 1 Central Statistical Office, Government of the Republic of Zambia, Lusaka, Zambia; 2 Global AIDS Program, Centers for Disease Control and Prevention, Government of the United States of America, Lusaka, Zambia; 3 CTS Global, Inc. Assigned to: Centers for Disease Control and Prevention, Lusaka, Zambia; 4 Futures Group/MEASURE Evaluation, North Carolina, USA

**Keywords:** cause of death, cause-specific mortality, mortality, verbal autopsy, Zambia

## Abstract

**Background:**

Verbal autopsy (VA) can be used to describe leading causes of death in countries like Zambia where vital events registration does not produce usable data. The objectives of this study were to assess the feasibility of using verbal autopsy to determine age-, sex-, and cause-specific mortality in a community-based setting in Zambia and to estimate overall age-, sex-, and cause-specific mortality in the four provinces sampled.

**Methods:**

A dedicated census was conducted in regions of four provinces chosen by cluster-sampling methods in January 2010. Deaths in the 12-month period prior to the census were identified during the census. Subsequently, trained field staff conducted verbal autopsy interviews with caregivers or close relatives of the deceased using structured and unstructured questionnaires. Additional deaths were identified and respondents were interviewed during 12 months of fieldwork. After the interviews, two physicians independently reviewed each VA questionnaire to determine a probable cause of death.

**Results:**

Among the four provinces (1,056 total deaths) assessed, all-cause mortality rate was 17.2 per 1,000 person-years (95% confidence interval [CI]: 12.4, 22). The seven leading causes of death were HIV/AIDS (287, 27%), malaria (111, 10%), injuries and accidents (81, 8%), diseases of the circulatory system (75, 7%), malnutrition (58, 6%), pneumonia (56, 5%), and tuberculosis (50, 5%). Those who died were more likely to be male, have less than or equal to a primary education, and be unmarried, widowed, or divorced compared to the baseline population. Nearly half (49%) of all reported deaths occurred at home.

**Conclusions:**

The 17.2 per 1,000 all-cause mortality rate is somewhat similar to modeled country estimates. The leading causes of death -- HIV/AIDS, malaria, injuries, circulatory diseases, and malnutrition -- reflected causes similar to those reported for the African region and by other countries in the region. Results can enable the targeting of interventions by region, disease, and population to reduce preventable death. Collecting vital statistics using standardized Sample Vital Registration with Verbal Autopsy (SAVVY) methods appears feasible in Zambia. If conducted regularly, these data can be used to evaluate trends in estimated causes of death over time.

## Background

Mortality is one of the most important indicators for measuring the health of the population in a country. But population-based causes of death have not been well described in many developing countries. Vital registration requires robust and systematic data collection, which is often difficult in these settings. Data on causes of death in most developing countries are incomplete and of poor quality, partly because most deaths are not attended by physicians or medically certified [1-3]. Only 12% of countries worldwide have high-quality mortality data from vital events registration, while 75 countries do not have any information on cause-specific mortality [4]. Less than a third of all deaths worldwide have causes that are medically certified [5]. Because of this, verbal autopsy (VA) methods are increasingly being used to ascertain the rate and leading causes of death, particularly in less-developed countries [6,7]. In Zambia, vital events registration is not robust or systematic and has failed to report on home deaths. Verbal autopsy methodology could become an important way to collect and report mortality data in Zambia [8-11].

Sample Vital Registration with Verbal Autopsy (SAVVY) is one method used to collect vital events data in regions where vital events registration is poor [12]. During a verbal autopsy, an interviewer trained in verbal autopsy methods asks the next of kin or caregiver open and structured questions about symptoms of the illness and events leading to the death. Specific symptoms reported are used to code causes of death using the 10^th ^revision of the International Classification of Diseases (ICD-10) [13]. Currently, the most common way to code symptoms into causes of death is by physician review. But, increasingly, computer-generated algorithms are being tested and validated for personnel-cost-free coding [11,14,15].

Standard World Health Organization (WHO)-recommended procedures suggest that cause of death be determined by administering verbal autopsy interviews using standard questionnaires after a baseline survey is conducted to identify deaths in a certain discrete period [16].

Other countries in the Southern African region have conducted VA studies using slight variations on the recommended WHO methodology. For example, Mozambique implemented a post-census mortality survey using VA methods [17], while other VA studies conducted in the region have focused on smaller communities [18,19].

Zambia has a population estimated at 13 million and is located in sub-Saharan Africa, bordering eight countries, Namibia, Angola, the Democratic Republic of the Congo, Tanzania, Mozambique, Malawi, Zimbabwe, and Botswana. The country is divided into nine provinces, within which there are 72 districts. Districts are further stratified into Census Supervisory Areas (CSAs) [20]. With the exception of Lusaka and Copperbelt provinces, Zambia is predominantly rural; an estimated 61% of the population resides in rural areas [21].

Information on mortality is collected by health facilities throughout Zambia. However, this system fails to collect data on home deaths, which are thought to represent a substantial proportion of deaths in the country [12]. Therefore, in spite of having the necessary regulatory framework that supports the maintenance of a vital statistics system in Zambia, the current system does not generate usable vital statistics. Mortality estimates have not been sufficiently reliable for setting health sector priorities or for assessing program progress and impact.

The goal of this study was to pilot implementation of a standardized process for collecting vital events data in Zambia. The objectives were to determine the feasibility of using SAVVY for this purpose and to estimate age-, sex-, and cause-specific mortality fractions for four pilot provinces in Zambia for a two-year period in 2009 and 2010.

## Methods

### Sampling

Data for SAVVY were collected by the Government of the Republic of Zambia's Central Statistical Office (CSO). SAVVY was implemented in four provinces from January to December 2010. We used the 2000 Zambia Census of Population and Housing data [20] as the sampling frame and selected a stratified one-stage random sample. In order to increase the efficiency of the sample design, the sampling frame of CSAs was divided into urban and rural strata that were as homogeneous as possible.

This pilot phase of SAVVY was conducted in 33 of 10,869 CSAs in Central, Luapula, Lusaka, and Southern provinces. The 33 CSAs were selected to represent different population densities and socioeconomic characteristics, and present various potential logistical challenges.

### Data collection

A baseline census was conducted in selected CSAs in January 2010 to count and describe the populations of the areas selected. VA interviews were conducted for all deaths reported to have occurred in the 12 months preceding the baseline census as well as for all deaths that occurred between January and December 2010. We carried out quarterly independent re-enumeration of populations to verify resident populations and death registration completeness as a quality measure [12]. Zambia used the standard and recommended WHO [22] VA questionnaires with slight adaptations to reflect the Zambian context (e.g., inclusion of a question on type of health facilities) for the collection of neonatal, child, and adult deaths and the causes of death. Characteristics collected included sex, age at death, marital status, education, place of death, health services utilization, rural or urban residence, and province. Nurses, other medical personnel, and, in some cases, teachers were trained as VA interviewers. VA Interviewers were assisted in the field by key informants whose duty was to inform the VA interviewers of every death that occurred in the CSAs in which they worked. Key informants, mostly community health workers and traditional birth attendants, were chosen from the CSAs in which SAVVY was implemented. MEASURE Evaluation's SAVVY methods are also described elsewhere [12].

### Coding

Nine physicians were trained on VA questionnaire review, how to produce a death certificate, and how to assign an immediate and underlying cause of death based on ICD-10 guidelines and coding principles. After the VA interviews were conducted, two physicians independently reviewed each VA questionnaire to determine a probable cause of death. They each completed a death certificate for the VA death and assigned an ICD-10 code. The death certificates and ICD-10 codes completed by the two physicians were then compared. If they agreed, the cause of death assigned was considered final. If they disagreed, they reviewed the VA questionnaire together to reach an agreement. If they failed to reach consensus on the underlying cause, the cause of death for that particular VA death was considered undetermined.

### Data analysis

SAVVY data for the four provinces were analyzed using Stata v11 (StataCorp LP, College Station, Texas). Characteristics of the population are presented along with mortality rates and cause-specific mortality fractions (CSMFs) by age and sex for the leading causes of death. CSMF refers to the proportion of deaths due to specific cause divided by the total number of deaths. For CSMF estimates, we aggregated and tabulated causes of death based on ICD-10 classification according to the WHO Tabulation List [13] adapted for Zambia. Additionally, we explored the type and place of services sought for medical care by those who died in the period leading to death (generally in the three months prior to death).

We used Pearson's chi-square tests to compare characteristics of those who died to the baseline census population and selected CSMFs between selected groups. We calculated the denominator for all-cause mortality by adding the number of individuals who were recorded as deceased in the 12 months preceding the survey to the total population in sampled areas from the census so that we had a complete census for the years we were recording deaths.

### Ethics

The protocol was approved by the Research Ethics Committee at the University of Zambia and the Zambian Ministry of Health. The Centers for Disease Control and Prevention (CDC) Institutional Review Board approved the evaluation as nonresearch.

## Results

Among the four provinces assessed, a total of 1,107 deaths were identified to have occurred during 2009 and 2010. During visits to the households to conduct VA interviews, 51 of these deaths were determined to have occurred prior to 2009, and were excluded from analysis. All subsequent summaries and analyses reflect the remaining 1,056 deaths.

A close adult relative (mother, father, sibling, or spouse) participated in the VA interview for 687 (65%) reported deaths, a child of the deceased participated for 96 (9%) deaths, other relatives participated for 259 (25%) deaths, and nonrelatives participated for 14 (1%) deaths. Of the total 1,056 deaths, 1,006 (95%) respondents reported that they had lived with the deceased in the period leading to death. An eligible respondent agreed to participate in the study for each death. There were no refusals.

The crude all-cause mortality rate was 17.2 per 1,000 (95% CI: 12.4, 22.0). More deaths occurred among males (584, 55%) than females (472, 45%) although slightly more females (51%) than males (49%) were reported in the dedicated census. The number of deaths in children under 5 years of age (365, 34%) was disproportionately high relative to this group's population (15%). Among the 70 (7%) neonatal (0 to 27 days) deaths, 40 (57%) were male and 30 (43%) were female. People who had a primary or no education (371, 57%) also contributed a disproportionately higher number of deaths than the population they represented (7,103, 41%). Marriage was as common in those who died as in the baseline census (approximately 50%). But those who had died were three to six times as likely to be widowed (115, 18%) or divorced (67, 10%) compared to the baseline population (5% and 3% respectively; Table 1).

**Table 1:** Socio-demographics of the deceased identified by SAVVY interview, and of the baseline population from the dedicated census

Characteristic	Deceased (N = 1056) n (%)	Baseline Census (N = 30,315) n (%)
Male sex	584 (55.3)	14,851 (49.0)

Age group		
0-4 yrs	365 (34.5)	4566 (15.1)
5-14	52 (4.9)	8400 (27.7)
15-49	430 (40.7)	15,542 (51.3)
50-64	95 (9.0)	1295 (4.3)
65+	114 (10.8)	512 (1.7)

Highest education level reported^1^		
None	82 (12.7)	556 (3.2)
Primary	289 (44.7)	6547 (37.7)
Secondary	195 (30.2)	7860 (45.3)
Higher	38 (5.9)	1954 (11.3)
Unknown	42 (6.5)	432 (38.0)

Marital status^1^		
Never married	119 (18.4)	6853 (39.5)
Married/Living with partner	322 (49.9)	8763 (50.6)
Widowed	115 (17.8)	928 (5.4)
Divorced	67 (10.4)	588 (3.4)
Separated	19 (2.9)	198 (1.1)
Unknown	4 (0.6)	19 (0.1)

Province		
Central	180 (17.1)	3310 (10.9)
Luapula	312 (29.6)	1210 (4.0)
Lusaka	348 (32.9)	14,268 (47.1)
Southern	216 (20.4)	11,527 (38.0)

^1^Among adults and teenagers only; Deceased: N = 646, Population: N = 17,349.

**Figure 1:**
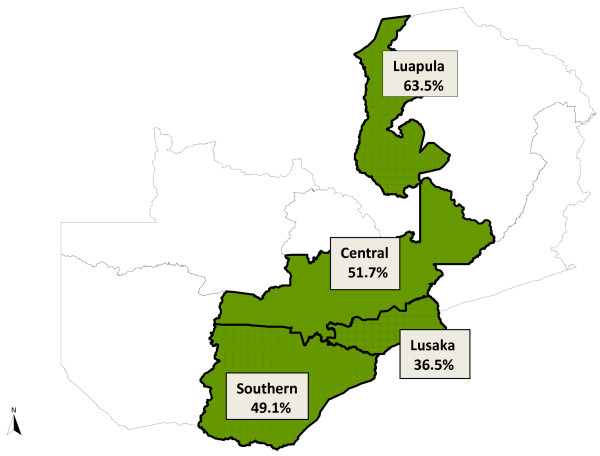
**Map of Zambia showing the percent of deaths reported to have occurred at home in 2009 and 2010 in each of the four pilot provinces**.

**Figure 2:**
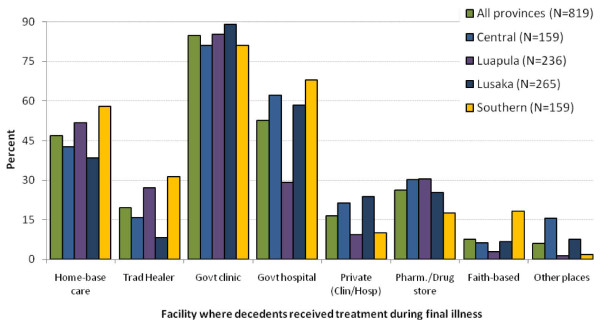
**Health care utilized by the deceased whose relatives reported that they received some type of treatment between final illness onset and death (n = 819)**. * The proportions across the different type of care/health facility sought do not add up to 100% because many individuals visited more than one type of health care/service during illness in the period leading to death. "Other places" includes hospices.

Of all reported deaths, 518 (49%) occurred at home. The place of death varied considerably among the provinces. In rural Luapula Province, approximately 64% (198) of reported deaths occurred at home, while in urban Lusaka, 37% (127) of deaths occurred at home (Figure 1). The majority (819, 77%) of the deceased sought some form of medical treatment in the period before death and many sought care at more than one facility (Figure 2). Most people (> 80%) who sought care went to a government clinic prior to death, and more than 53% sought treatment at a government hospital. Only 30% of the deceased in rural Luapula, but approximately 68% in rural Southern Province, were reported to have sought treatment at a government hospital. Overall, more than 46% of the deceased received home-based care.

**Figure 3:**
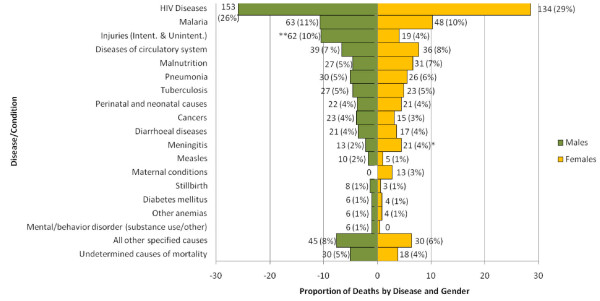
**Causes of death for males and females of all ages from all deaths recorded by Zambia's SAVVY (N = 1056) from 2009-2010**. *p < 0.05. **p < 0.001 Note: All other specified causes include the remainder of infectious and parasitic diseases, diseases of the liver, other digestive system disorders, other CNS disorders, other chronic obstructive pulmonary diseases, disorders of the kidney, other mental and behavioural disorders, asthma, senility/oldage, sickle-cell disorders, other respiratory diseases, duodenal ulcer, hernias, disorders of the skin and subcutaneous tissue, other diseases of the urinary system, other disorders of the genital organs, abdominal pain, Sudden Infant Death Syndrome, leprosy, viral hepatitis, other blood diseases, epilepsy, diseases of the oesophagus, stomach and duodenum, hyperplasia of prostrate, stomach and other digestive system disorders.

The leading causes of death were HIV/AIDS (287, 27%), malaria (111, 10%), injuries and accidents (81, 8%), diseases of the circulatory system (75, 7%), malnutrition (58, 6%), pneumonia (56, 5%), and tuberculosis (50, 5%) (Figure 3). The remaining deaths were caused by perinatal and neonatal causes, diarrheal diseases, cancers, meningitis, maternal conditions, measles, stillbirth, other anemias, diabetes mellitus, and mental and behavioral disorders due to substance use and other causes. Combined, other specified conditions (including the remainder of infectious and parasitic diseases, diseases of the liver, other digestive system disorders, mental disorders, stomach and other digestive system disorders) represented approximately 8% (75) of the total causes of death while 5% (48) had causes that were undetermined (Figure 3).

Cause-specific mortality fractions varied by disease and gender. Males died most often of HIV/AIDS, and most (125, 82%) HIV deaths among males occurred in those over 15 years of age. Malaria, injuries (transport related, drowning, falls, exposure to smoke, fire and flames, accidental poisoning, and assault), and diseases of the circulatory system were the other leading causes of death for males. Men died of injuries more often than women did (10% vs. 4%, p < 0.01; Figure 3).

The leading causes of death for females were HIV/AIDS, malaria, diseases of the circulatory system, and malnutrition (Figure 3). Most (105, 78%) females dying of HIV/AIDS deaths were over 15 years of age. Approximately 93% of the deaths in females due to malnutrition occurred in children and young teens between 4 weeks and 14 years of age. Meningitis caused a statistically significantly greater number of deaths in girls aged 4 weeks to 14 years vs. boys in the same age group (7% vs. 2%, p < 0.05). Maternal conditions also contributed to 13 (3%) of deaths among females. Although not statistically significant, more male infants died of stillbirth than females (1.5% vs. 0.6%, p < 0.25).

## Discussion

All-cause crude mortality was 17.2 per 1,000 person-years in the four provinces studied from 2009 to 2010; nearly half (49%) of deaths occurred at home. Overall leading causes of death were HIV/AIDS, malaria, and circulatory diseases, generally reflecting the order of sex-specific causes. Sex-specific differences in the order included injuries, the third leading cause of disease for men, and malnutrition, the fourth leading cause of death for women.

These leading causes of death were similar to those previously reported in the region [18,23,24]. In a worldwide summary of mortality published in 2009, leading causes of adult mortality in the African region were derived largely from population-based studies, and included HIV/AIDS (35%), other infectious causes (including malaria), and injuries (for men) [22]. Separate SAVVY-based studies in Tanzania and Kenya showed that the majority of non-infant deaths were attributable to HIV/AIDS, tuberculosis, and malaria, and approximately 6% were attributable to cardiovascular disease (CVD).

With modification based on lessons learned from this pilot, new technologies being developed, and a gradually increasing governmental commitment to fund collection of vital statistics data, it is feasible that vital registration data may be collected using SAVVY methodology in the future.

Zambia was the first country in Africa to use WHO-recommended SAVVY methodology to collect vital events data. WHO recommends conducting a dedicated census just prior to conducting verbal autopsy interviews. These activities require donor funding and the undivided attention of professional government staff. While costly on monetary, opportunity cost, and staffing bases, this rigorous methodology allows for collection of standardized census data specific for vital events registration. Adaptations of WHO-recommended SAVVY methodology that rely on national censuses, like the one conducted in Mozambique in 2007 [17], likely realize cost and time efficiencies that may improve sustainability. However, these efficiencies must be weighed against the adverse impact that longer recall periods may have on data quality [6,25]. Using a dedicated census allows for a shorter recall period for retrospective identification of deaths. Additionally, the recall period can be determined by the study implementers, rather than by scheduled national censuses. Using standardized approaches to verbal autopsy and SAVVY-based vital registration could also improve the ability to compare results across countries and regions [16].

Zambia employed physicians to code interviews into ICD-10 causes of death (physician- certified verbal autopsy [PCVA]). Two physicians coded each interview and, if their codes differed, they discussed the case to agree on a final code. Physicians were unable to consider medical records in coding deaths as recommended by WHO because most families of those who had died did not keep these medical records. Despite this limitation, we viewed PCVA and duplicate coding as strengths. However, recent publications have suggested a low (30%) concordance between PCVA and a gold standard, in this case known cause of death [26]. Recent reports have also cast doubt that duplicate coding improves data quality [27,28].

Computer-based algorithms such as InterVA and the Symptom-Pattern and the newly developed Tariff and Random Forest methods are promising alternatives to PCVA [29-31]. Algorithmic methods have been shown to perform as well as or better than PCVA in cause of death assignment without the personnel cost [29,30,32]. For particular individual causes of death, some algorithms have been shown to perform better than PCVA. But algorithms lack the ability to identify and prioritize causes of death that are of public health importance in specific settings, to adapt to changing disease patterns, and to accurately identify less common causes of death [28,30]. Overall, in recent comparisons, despite statistical differences in results generated by PCVA and algorithm-coded methods, leading causes of disease and groups most burdened have been similar and have had the same policy implications [19,28].

During data collection for this pilot phase, we did not use algorithms to code causes of death because they have only recently been developed and validated. Once they are refined and made available for tailoring and testing in Zambia, they could be used here.

Although this was a pilot and included just four of the nine provinces in Zambia, our crude all-cause mortality rate, 17.2 per 1,000 person-years, was somewhat similar to the 13.3 per 1,000 person years estimated for 2009 and 2010 by the Central Statistics Office's (CSO) "Population Projections Report" based on projections from 2000 census data [33]. Our estimate, which included a substantial proportion of residents of Lusaka Province, was essentially equal to CSO's projections for Lusaka Province (17.1 per 1000) and similar to results from a recently conducted analysis reporting 14.1 to 14.5 deaths per 1,000 in Lusaka Province [34]. Although samples were not drawn to be representative of the country, our crude maternal mortality rate (1.6 per 100,000 women aged 15-49) was somewhat similar to the 1.2 per 100,000 rate reported by the Demographic Health Survey (DHS) for 2002-2003 [35]. However, our under-5 mortality was substantially lower (80 per 1,000) than the DHS reported with regard to 2003-2004 data (119 per 1,000) [35]. This difference could suggest an ascertainment gap in our data that should be investigated. Under-5 mortality could also be underreported because of stigma associated with discussing early childhood deaths. Otherwise, few sources of mortality data exist in Zambia. To our knowledge, there is no other source of representative data on the distribution and causes of death in Zambia.

More than 80% of people who subsequently died were reported to have sought treatment at a government clinic at some time prior to death. Many delayed too long. These findings suggest that educational material should be posted in waiting areas of government health clinics to alert people to *early *signs and symptoms of illnesses that should prompt them to seek medical care. Based on the demographic profile of those who died, messaging should be simple, pictorial, and in large print.

Despite rapid scale-up of national programs to provide free highly active antiretroviral treatment (HAART) and prevention of mother-to-child transmission, HIV is still the leading cause of death in these four provinces. This finding is similar to other countries in sub-Saharan Africa [24,28] and the entire African region [23]. Research suggests that HIV-related death is most common in the three months following treatment initiation and is associated with advanced HIV disease at presentation [36], thought to indicate delays in seeking care. Long distances from homes to health care centers providing HAART have been linked to delayed treatment, particularly in rural areas [37]. Zambia is currently incorporating new, more aggressive treatment guidelines that may improve survival [38]. It is hoped that implementation of these guidelines will lead to reductions in HIV-related mortality, although they do not address the distance barrier. Zambia should be able to evaluate trends in HIV-related mortality before and after implementation of the new guidelines with the continued and ongoing collection of vital events data using SAVVY.

Other leading causes of death reported here were also reported by others in the region, including malaria [24,39-41], circulatory diseases [23], and injury [23,42].

Challenges existed with data collection and analysis that should be taken into account when interpreting the data. First, verbal autopsy interviews were conducted by nurses who could have introduced their own professional judgment and biases into the coding process by selecting keywords associated with the illnesses that they inadvertently "diagnosed" during the interview. Secondly, the census did not collect any information about ages under 1, so it was not possible to calculate neonatal mortality rates. Thirdly, when field staff assessed health care sought, they meant in the three months prior to death, but we understand that this time frame was not consistently explained. Because of this oversight, in addition to health care sought for the illness leading to death, we may have also captured health care sought for conditions that the person had previously but from which they didn't die. Fourth, this was the pilot phase of SAVVY in Zambia and the sampled areas do not necessarily represent the country. The next phase is designed to complement the pilot and, together, provide nationally-representative estimates. Fifth, most of the households interviewed were unable to provide clinical records such as laboratory results. Our reliance on a lay description of the family member's symptoms likely resulted in misclassification of cause of death in some cases. Sixth, as a cultural practice, stillbirths and neonatal deaths are not generally acknowledged by families as deaths, and so are likely greatly undercounted in this study. Seventh and finally, sample randomization for this 2009-2010 assessment was based on a national census from 2000, which was clearly out of date.

Other aspects of Zambia's application of SAVVY likely contributed to high quality and completeness of data. For instance, a dedicated census allowed for shorter recall periods for our interviewees. Additionally, community health workers and traditional birth attendants were employed and trained to identify deaths in their own communities for autopsy interview. As community members, they also facilitated entry of SAVVY interviewers into their neighbors' households.

In part, because of the strengths and despite the weaknesses, these data can be used to determine needs and gaps in the health care system. Results could be used to develop community-based interventions to improve survival in the groups identified as most at risk for death. Based on our results, interventions could include improvements in HAART access for people with HIV; access to treated mosquito nets for malaria prevention and access to prompt treatment for those with malaria; clinically-attended birthing and nutritional support for females; access to information about preventing and treating circulatory diseases; and increased education to parents about knowledge of signs and symptoms that should prompt urgent medical attention of their young children. Further in-depth study is needed to develop interventions to avert deaths, especially those that are preventable and treatable.

While SAVVY data have not yet been linked with Zambia's national electronic health records (EHR) system, "SmartCare," identifiers used in the system are compatible with those used in SAVVY. With dedicated effort, appropriate approvals, and confidentiality protections, the health care, morbidity, and mortality data collected in health facilities that are captured in SmartCare could be linked with the verbal autopsy and census data captured in SAVVY. This linkage could identify clinical antecedents of mortality, providing a more comprehensive description of gaps in care and prevention.

Finally, discussion is also needed worldwide to determine whether the gains enjoyed from standardizing SAVVY methods across countries are worth the potential loss of cost efficiency and perhaps the additional sustainability gained by integrating it with other activities.

## Conclusion

Results from this pilot study indicate that collection of verbal autopsies to estimate causes of mortality using SAVVY methods is feasible in Zambia. The SAVVY methodology enabled Zambia, a country where half of all deaths occur at home, to collect field-based vital records data and report causes of death in selected provinces for the first time. Lessons learned from Zambia's pilot of SAVVY and the experiences of other countries will enable us to modify our methods with an aim of improving data quality and sustainability of SAVVY. Zambia hopes to incorporate SAVVY into national strategic plans so that this essential vital statistics information can be used to identify gaps in quality and access to health care and identify shortfalls and successes of interventions.

## Competing interests

The authors declare that they have no competing interests.

## Authors' contributions

PS, SK, and RM participated in the conception and design of the study. SSM, SK, RM, MC, PS, and MM participated in the analysis of the results. RM and MC wrote the statistical code and generated the computer output. SSM, SK, MC, RM, and MM drafted the paper. WM and DP reviewed and commented on the manuscript, and WM provided technical and managerial support for the Central Statistics Office authors. All authors read, contributed to, and approved the manuscript.
